# Assessment of Physical Activity and Sleep Quality Among Doctors and Medical Students: A Cross-Sectional Study From South India

**DOI:** 10.7759/cureus.65807

**Published:** 2024-07-30

**Authors:** Jamunarani Srirangaramasamy, Venkatesh Karthikeyan, Raghunandan Ramanathan, Abul Hasan KM, Basavaraj SY, Naveen Suthar, Hariharan Sathananthan

**Affiliations:** 1 Pathology, Bhaarath Medical College and Hospital, Chennai, IND; 2 Community and Family Medicine, All India Institute of Medical Sciences, Patna, Patna, IND; 3 General Practice, Tagore Medical College and Hospital, Chennai, IND; 4 Pediatric Surgery, City Hospital, Erode, IND; 5 Internal Medicine, H2 Clinic, Chennai, IND

**Keywords:** south india, cross-sectional study, medical students, doctors, physical activity, sleep quality

## Abstract

Introduction: Sleep quality is critical for medical professionals and students, who often face sleep disturbances due to demanding schedules. This study explores the association between physical activity and sleep quality among doctors and medical students in Tamil Nadu, India, addressing a notable gap in existing research.

Methods: This cross-sectional study was conducted in Tamil Nadu, India, targeting doctors and medical students. Participants were recruited through an online Google Forms (Google LLC, Mountain View, California, United States) questionnaire distributed via social media platforms, which included the International Physical Activity Questionnaire (IPAQ) and the Pittsburgh Sleep Quality Index (PSQI) which provided measures of physical activity in MET (Metabolic Equivalent of Tasks) minutes/week and comprehensive sleep quality assessments, respectively, with a PSQI score above 5 indicating poor sleep quality. Data analysis was performed using jamovi version 2.2.2.

Results: In this study of 222 participants, predominantly young adults (80% were aged 18-40) and medical students (68.6%), we found a high prevalence of poor sleep quality (74.9%). Physical activity levels varied, with only 5.4% engaging in high levels of activity and no significant association between physical activity level and sleep quality (p=0.659). Additionally, while males showed significantly higher MET scores than females (p < 0.001), there was no significant correlation between MET scores and PSQI scores (p=0.205, r=-0.075).

Conclusion: This study highlights a high prevalence of poor sleep quality and a low prevalence of high physical activity among medical professionals and students in South India. The study findings underscore the need for targeted interventions to enhance sleep quality and overall well-being in this demographic.

## Introduction

Sleep quality is critical to overall health and well-being, particularly in medical students and doctors. Most medical students and doctors have poor sleep quality, insomnia, and stress due to irregular schedules and the demanding nature of their profession and education [1-3]. Poor sleep quality not only affects cognitive function and mood but also increases the risk of various physical and mental health problems, including cardiovascular disease, obesity, diabetes, depression, and anxiety [4]. Due to the highly demanding nature of their profession and education, medical students and doctors also tend to be less physically active [4-6]. While the importance of adequate sleep for medical professionals and students is well recognized, there is limited research specifically examining the association between physical activity levels and sleep quality within this population, particularly in the context of South India. Understanding this relationship is essential for planning targeted interventions and strategies to promote better sleep habits and overall health among doctors and medical students in this region.

Physical activity has been consistently associated with improved sleep quality in the general population, with studies suggesting that regular exercise can enhance sleep duration, efficiency, and subjective perceptions of sleep [7,8]. However, the extent to which this association holds true among medical professionals and students remains unclear. Given this population's unique challenges and stressors, exploring the relationship between physical activity and sleep quality is crucial for identifying potential avenues for intervention. By conducting a cross-sectional study examining the association between physical activity levels and sleep quality among doctors and medical students, we aim to address this knowledge gap and provide valuable insights into the factors influencing sleep health in this population.

Findings from this study will aid in developing targeted interventions, such as promoting regular physical activity to improve sleep quality and overall well-being among medical professionals and students. Ultimately, enhancing sleep quality among doctors and medical students can have profound implications for their performance, job satisfaction, and long-term health outcomes, benefiting individuals and healthcare systems [9].

Hence, the objective of this study is to assess the quality of sleep and level of physical activity among doctors and medical students from Tamil Nadu, South India. Further, the possible association between sleep quality and physical activity will be explored. Despite extensive search, no published literature could be found on the association of physical activity with sleep quality among doctors and medical students. To the best of our knowledge, this will be the first study in this geographical region to assess the physical activity and sleep quality among doctors and medical students.

## Materials and methods

Study setting and population

The study was conducted in Tamil Nadu, a region distinguished by its high concentration of medical educational institutions, with 74 medical colleges, the highest number in India [10]. The study participants comprised doctors and medical students who reside in Tamil Nadu and ranged in age from 18 to 69 years. Inclusion criteria included current enrollment or employment in a medical institution in Tamil Nadu and consent to participate. Those who did not consent to participate were excluded from the study. The study was approved by the Institutional Ethics Committee, Bhaarath Medical College and Hospital, Chennai, Tamil Nadu (approval number: BIEC-056-23, dated July 20, 2023).

Study design, duration, and sampling technique

A cross-sectional study design was adapted for this study, and the data collection period spanned three months, from November 2023 to January 2024. Convenience sampling technique was employed to recruit study participants. An online Google Forms (Google LLC, Mountain View, California, United States) form distributed via social media platforms (e.g., WhatsApp, Facebook; Meta Platforms, Inc., Menlo Park, California, United States) was used to recruit participants and facilitated the collection of responses, allowing for a rapid and broad reach across the target population.

Sample size calculation

According to a study conducted by Palo and Das, 79.2% of the professionals in India had poor sleep quality [11]. Assuming that 79% of our study participants will have poor sleep quality, and applying a design effect of 2, the sample size for the study was calculated to be 205, for estimating the expected proportion with 10% relative precision and 95% confidence interval (CI).

Data collection and study tool

Data was collected using a self-administered online form, which included well-structured and validated questionnaires: the International Physical Activity Questionnaire - Short Form (IPAQ) [12] and Pittsburgh Sleep Quality Index (PSQI) [13]. The questionnaire was divided into three sections. The first section gathered sociodemographic details of the participants, such as age, gender, college affiliation, and residence. The second section assessed physical activity levels using the IPAQ, and the third section evaluated sleep quality using the PSQI. The questionnaire was pilot-tested with a small group of medical students (n=20) to ensure clarity and functionality. This approach ensured comprehensive data collection on the target population's physical activity and sleep quality.

The IPAQ is widely used for assessing adult physical activity levels. It consists of seven questions about the frequency and duration of moderate and vigorous physical activities and walking in the last seven days. The questionnaire also includes items on sedentary behavior and sitting time. Responses were used to calculate total physical activity levels in metabolic equivalent of tasks (MET) minutes per week [14]. The IPAQ provides a quick and reliable way to assess physical activity levels across different populations, making it valuable for research and public health initiatives to promote physical activity and understand its impact on health outcomes [14].

The PSQI is a widely used tool for assessing adult sleep quality. It consists of a self-report questionnaire evaluating various aspects of sleep over the past month. The questionnaire comprises 19 items grouped into seven components: subjective sleep quality, sleep latency, sleep duration, sleep disturbances, use of sleep medication, habitual sleep efficiency, and daytime dysfunction. Each component is scored on a scale from 0 to 3, with higher scores indicating poorer sleep quality. The total score ranges from 0 to 21, with scores above 5 indicating poor sleep quality. The PSQI provides a comprehensive assessment of sleep patterns and disturbances, making it valuable for both clinical and research purposes in understanding and addressing sleep-related issues.

Statistical analysis

Scores were assigned to assess levels of physical activity and sleep quality. A cut-off of 5 was used to differentiate between good and poor sleep quality, where a PSQI score greater than 5 implies poor sleep quality [15]. Study participants were categorized into three groups based on IPAQ score: low physical activity (<600), moderate physical activity (600 to 3000), and high physical activity (>3000) [14].

Data were entered into a Microsoft Excel sheet (Microsoft Corporation, Redmond, Washington, United States) and analyzed using jamovi version 2.2.2 (The jamovi project, 2021, https://www.jamovi.org). Results were tabulated or represented as figures where necessary. Descriptive analysis was done for sociodemographic variables such as age, gender, residence, and designation. Continuous variables, including age, IPAQ scores, and PSQI scores, were described using the mean and standard deviation (SD) or median and interquartile range (IQR), depending on the normality of the data distribution. Categorical variables such as gender, designation, proportion of physically inactive participants, and proportion of poor sleep quality, were expressed as frequencies and percentages. Pearson's test was used to determine the correlation between physical activity and sleep quality. Statistical significance was set at p < 0.05.

## Results

**Figure 1:**
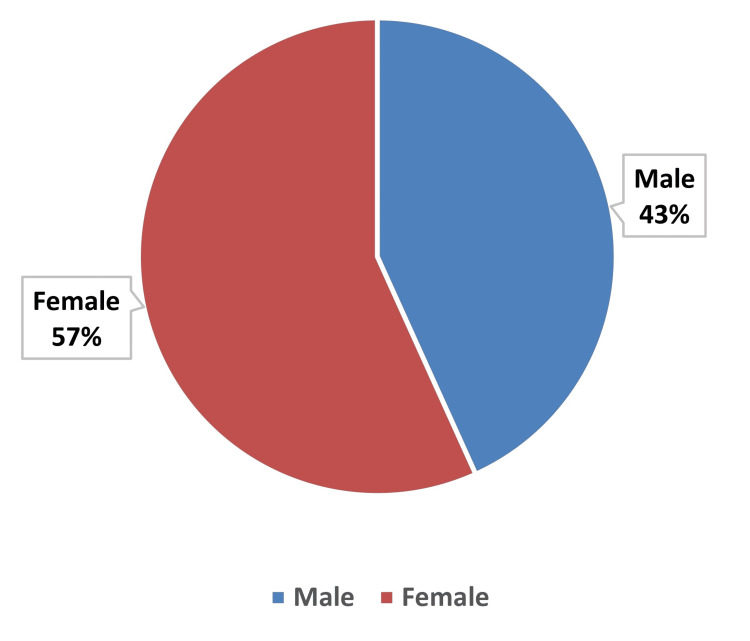
Gender-wise distribution of study participants (N=222)

The total number of participants was 222 (N). Most study participants were female (n=126, 57%), while the remaining were male (n=96, 43%) (Figure 1). This indicates a slightly higher representation of females in the study population.

**Figure 2:**
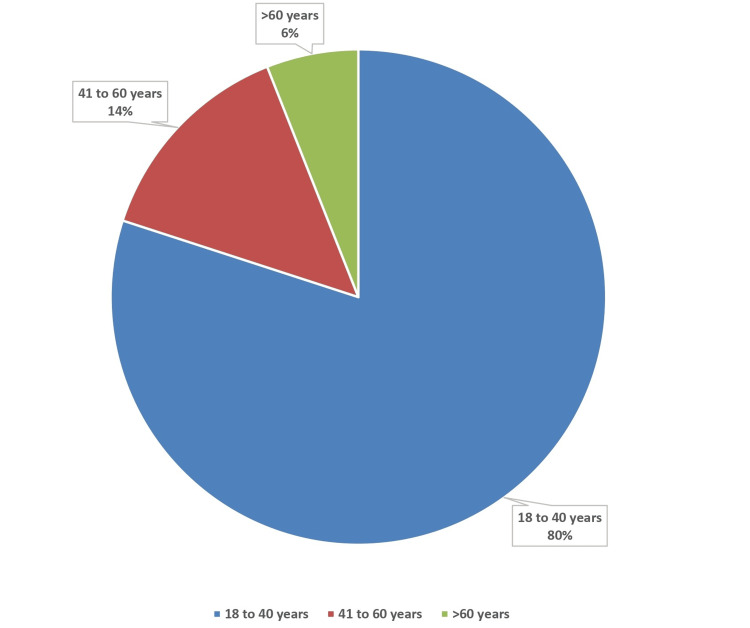
Age category-wise distribution of study participants (N=222)

The majority of the study participants (n=178, 80%) belonged to the age group of 18-40 years (Figure 2). This was followed by the age groups of 41-60 years (n=31, 14%) and >60 years (n=13, 6%).

**Figure 3:**
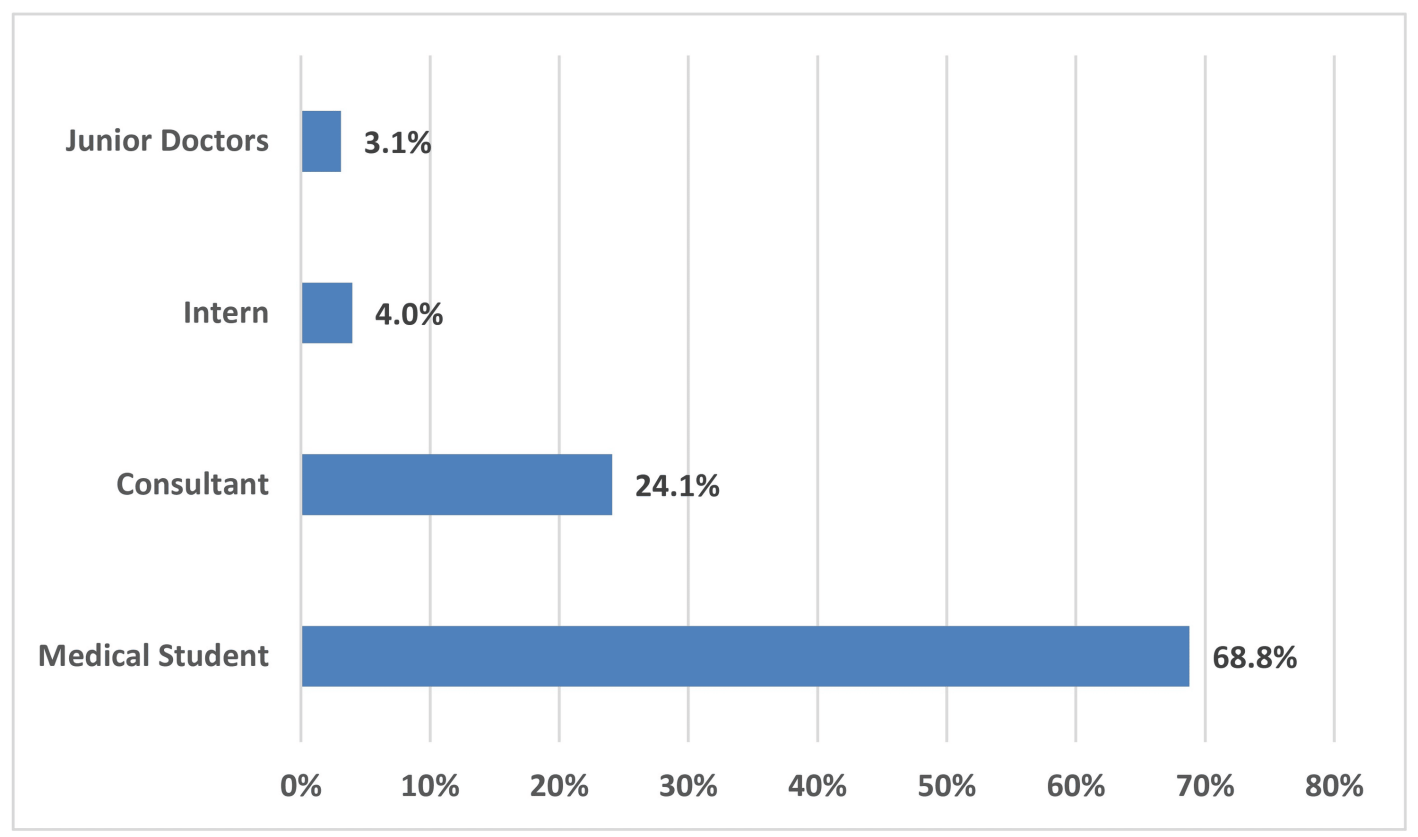
Distribution of study participants according to designation (N=222)

Most of the study participants were medical students (n=163, 68.8%). This was followed by consultants (n=54, 24.1%) and interns (n=9, 4%). Junior doctors accounted for the smallest proportion (n=7, 3.1%) (Figure 3).

The overall mean global PQSI score was 7.77 (±3.07). For males, the mean PSQI score was slightly higher at 7.96 (±2.91) compared to females, who had a mean score of 7.63 (±3.18). The mean difference in PSQI score between males and females was 0.328, which is not statistically significant, as shown by student’s t-test (t = 0.791, df = 221, p-value = 0.430) (Table 1). 

**Table 1:** Physical activity and sleep quality among the participants (N=222) *Student’s t-test, #Welch’s t-test PSQI: Pittsburgh Sleep Quality Index; MET: Metabolic Equivalent of Tasks

Variable		n	Mean ± SD	p -Value
Global PSQI score	Total	222	7.77±3.07	0.43*
	Male	96	7.96±2.91	
	Female	126	7.63±3.18	
Total MET score	Total	222	1259±922	<0.001^#^
	Male	96	1602±971	
	Female	126	1000±793	

The overall mean MET score was estimated to be 1259 (±922). Males reported a significantly higher mean MET score of 1602 (±971) compared to females, who had a mean score of 1000 (±793). The mean difference between both genders was 602, which was statistically significant, as shown by Welch’s t-test (t = 4.951, df=180, p-value = <0.001). This indicates that there was a higher level of physical activity among males. Welch’s t-test was applied in the latter case as the assumption of homogeneity of variance was not met. 

These results suggest significant gender differences in physical activity levels, as indicated by MET scores, but no significant gender differences in sleep quality, as measured by the PSQI scores.

With regard to sleep, out of 58 participants engaging in low physical activity, 12 (20.7%) reported good sleep quality, while 46 (79.3%) reported poor sleep quality. Among 153 participants with moderate physical activity, 41 (26.8%) experienced good sleep quality, compared to 112 (73.2%) who experienced poor sleep quality. Of the 12 participants who engaged in high physical activity, three (25.0%) had good sleep quality, whereas nine (75.0%) had poor sleep quality (Table 2).

**Table 2:** Association between level of physical activity and sleep quality among study participants (N=222)

Physical Activity Level		Good Sleep Quality	Poor Sleep Quality	Total
Low physical activity	Observed	12	46	58
	% within row	20.7 %	79.3 %	100.0 %
Moderate physical activity	Observed	41	112	153
	% within row	26.8 %	73.2 %	100.0 %
High physical activity	Observed	3	9	12
	% within row	25.0 %	75.0 %	100.0 %
Total	Observed	56	167	223
	% within row	25.1 %	74.9 %	100.0 %

Overall, the percentages of good sleep quality across different levels of physical activity were relatively similar, with approximately one-quarter of participants in each category reporting good sleep quality (20.7% in low, 26.8% in moderate, and 25.0% in high). However, the majority of participants in each physical activity category reported poor sleep quality, making up 74.9% of the total cohort. These findings suggest a consistent pattern of poor sleep quality regardless of physical activity level among the study participants.

**Figure 4:**
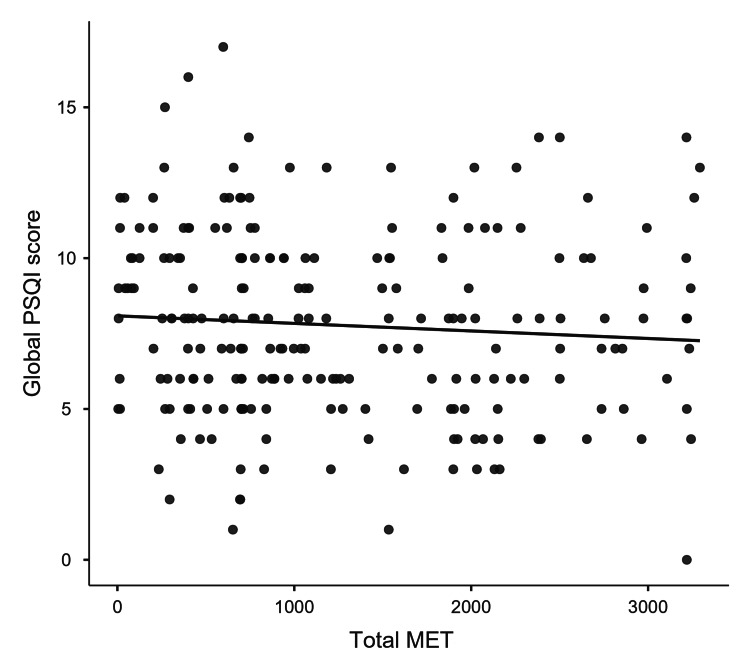
Correlation between level of physical activity and sleep quality among the study participants (N=222) PSQI: Pittsburgh Sleep Quality Index; MET: Metabolic Equivalent of Tasks

A Pearson's product-moment correlation was run to assess the relationship between the total MET score and the global PSQI score. The points were distributed across a range of MET values from 0 to over 3000, showing varying levels of physical activity among the participants (Figure 4). These scores, which assessed sleep quality, predominantly ranged between 5 and 10, with a few outliers approaching and possibly exceeding a score of 15. The scatter plot showed the relationship to be linear and negative with both variables, no outliers, and normally distributed, as shown by the Q-Q plot. The correlation between MET score and PSQI score was not significant (p-value 0.205, r = -0.075). The lack of a distinct downward or upward trend along the line of best fit implies that increases in physical activity do not correspond with predictable improvements or declines in sleep quality within the study participants.

## Discussion

This study aimed to evaluate sleep quality and physical activity among doctors and medical students in Tamil Nadu. Our results indicate a high prevalence of poor sleep quality among participants (74.9%), aligning with similar findings from Almojali et al. [2], who reported a 76% prevalence of poor sleep quality in their study. Contrary to existing literature that often shows a positive impact of physical activity on sleep [5], our study found no significant association between physical activity levels and sleep quality.

A closer examination of physical activity data revealed that most participants engaged in only moderate levels of physical activity, with very few reaching high activity levels. This pattern may reflect the limited time available for exercise due to the heavy workload and long hours associated with medical training and professional responsibilities [6]. Despite these moderate levels of activity, they did not translate into improved sleep quality, suggesting that the intensity or type of physical activity may not be sufficient to offset the high stress and poor sleep hygiene common among medical students and professionals.

Interestingly, our results did not align with those of Palo and Das [11], who reported significant improvements in sleep quality among professionals engaging in higher physical activity. This discrepancy could be due to differences in the cultural, geographical, and professional contexts between the populations studied. For instance, the majority of our study participants were younger medical students who might experience unique academic and social stressors affecting their sleep, independent of their physical activity levels.

Moreover, gender differences observed in physical activity levels, where males were more active than females, did not influence the overall poor sleep quality, suggesting that other factors such as psychological stress, academic pressure, and dietary habits play more substantial roles in influencing sleep quality among this population. These findings underscore the complexity of sleep quality determinants and highlight the need for a multifaceted approach to addressing sleep disturbances among medical professionals.

Our study used validated instruments like the IPAQ and PSQI, enhancing the reliability of our findings. Additionally, this study was the first to explore the association between physical activity and sleep quality among doctors and medical students in Tamil Nadu, India, providing novel insights into this population. The relatively large sample size of 222 participants further strengthens the study's statistical power and the generalizability of its findings within the region.

Despite its strengths, this study has several limitations that should be considered. The use of convenience sampling may limit the generalizability of the findings, as this method might not yield a sample representative of the broader population of medical professionals and students. Future studies could benefit from employing random sampling techniques to ensure a more representative sample. The cross-sectional design of the study precludes any causal inferences between physical activity and sleep quality; thus, longitudinal studies are needed to better understand these relationships over time. Additionally, the reliance on self-reported data introduces potential biases such as recall bias and social desirability bias, which could affect the accuracy of the reported data. Utilizing objective measures of physical activity (e.g., accelerometers) and sleep quality (e.g., polysomnography) in future research could mitigate these biases.

## Conclusions

Our study highlights a critical need for targeted interventions aimed at improving sleep quality among doctors and medical students. These interventions could benefit from also addressing physical activity but should consider the complex and possibly confounded relationships with other lifestyle and occupational factors. Future research could explore these relationships longitudinally with a larger and more diverse sample to tease out the nuanced impacts of physical activity on sleep quality and to identify other contributing factors to poor sleep quality in this population. Policymakers and healthcare administrators should consider implementing wellness programs that address both physical activity and mental health to enhance the overall well-being of medical professionals and students in the region.
